# TeleGlaukos: A Clinician-Led Adaptive Telemonitoring Platform for Hybrid Glaucoma Care

**DOI:** 10.3390/bioengineering13091090

**Published:** 2026-09-20

**Authors:** Jeniffer Jesus, Pedro Cardoso-Teixeira, João Chibante Pedro, Dália Meira, Ignácio Rodriguez-Uña, João Melo Beirão, Luís Bastião Silva

**Affiliations:** 1 Department of Ophthalmology, Unidade Local de Saúde de Entre Douro e Vouga, 4520-211 Santa Maria da Feira, Portugal; joao.chibante@ulsedv.min-saude.pt; 2 Department of Ophthalmology, Unidade Local de Saúde Gaia e Espinho, 4434-502 Vila Nova de Gaia, Portugal; daliamartinsmeira@gmail.com; 3 Department of Ophthalmology, Instituto Oftalmológico Fernàndez-Vega, 33012 Oviedo, Spain; ignaciorodriguezu@gmail.com; 4 Department of Ophthalmology, Unidade Local de Saúde Santo António, 4099-001 Porto, Portugal; brandaobeirao@gmail.com; 5 BMD Software, 3830-352 Aveiro, Portugal; lbastiao@gmail.com; 6 IEETA/DETI, University of Aveiro, 3810-193 Aveiro, Portugal

**Keywords:** telemonitoring, glaucoma, digital health, perioperative care, adaptive questionnaires, remote patient monitoring, system usability scale, ophthalmology

## Abstract

Glaucoma requires continuous surveillance, sustained treatment adherence, and timely recognition of postoperative or treatment-related complications. Conventional outpatient follow-up provides structured clinical assessment but offers limited visibility of symptoms between appointments. This study describes TeleGlaukos, a clinician-led adaptive telemonitoring platform designed for hybrid perioperative and therapeutic glaucoma care. Its principal innovation is a configurable protocol engine that transforms static symptom forms into patient-specific workflows through conditional questions, symptom-triggered branching, medication reminders, rule-based alerts, and bidirectional clinician–patient communication. The platform was implemented as a Progressive Web App connected to a modular Django backend through RESTful APIs and WebSocket-enabled communication. The clinical pilot cohort comprised 30 glaucoma patients followed between May 2025 and April 2026. The wider platform environment contained 95 registered accounts: 30 glaucoma patients, 10 ophthalmologists, 10 research or administrative users, 20 development and testing accounts, and 25 non-pilot registered users. Across the platform, 737 engaged sessions, 1329 events, 30 bidirectional messages, and 186 questionnaire responses were recorded. Structured monitoring identified suspected treatment-related adverse reactions in two patients, prompting earlier clinical reassessment and treatment adjustment. Usability was assessed in 22 respondents (18 patients and 4 ophthalmologists), yielding a mean System Usability Scale score of 79.43 ± 17.98 (95% CI: 71.46–87.40), corresponding to a Good adjective rating and an Acceptable usability range; internal consistency was high (Cronbach’s alpha = 0.882). The artificial intelligence-assisted module remained exploratory and was not evaluated as an autonomous clinical component. TeleGlaukos demonstrated technical feasibility, favourable perceived usability, and the capacity of adaptive symptom workflows to surface clinically relevant signals between visits. Larger multicentre studies are required to evaluate effectiveness, safety, adherence, workflow burden, and cost-effectiveness.

## 1. Introduction

Glaucoma is a leading cause of irreversible visual impairment and requires long-term surveillance to prevent progressive optic nerve damage and visual field loss [1]. Effective management depends not only on intraocular pressure control but also on continuous assessment of symptoms, treatment adherence, and postoperative recovery, particularly after surgical or therapeutic changes [2].

Traditional glaucoma follow-up is usually based on scheduled outpatient visits, supplemented when necessary, by telephone contact or unscheduled urgent care. Although clinically established, this model may produce fragmented information flows between visits and can delay recognition of patient-reported deterioration or adverse treatment effects. In addition, repeated hospital attendance may impose substantial practical burdens on patients and caregivers, while outpatient referral patterns may also contribute to pressure on ophthalmology services [3,4,5].

Digital health and telemedicine approaches have increasingly been proposed to improve continuity of care in chronic diseases. In glaucoma, recent developments include home tonometry, remote perimetry, digital imaging, and hybrid models of specialist follow-up [6,7,8,9]. Nevertheless, many generic telehealth solutions lack disease-specific workflow logic, configurable clinical protocols, structured symptom escalation, and sustained clinician–patient communication. Glaucoma follow-up therefore requires not only remote measurement but also longitudinal symptom surveillance, medication adherence support, and clear pathways for clinical reassessment.

TeleGlaukos was developed as a clinician-led, web-based telemonitoring platform to address these needs. Rather than reproducing a conventional consultation online or focusing exclusively on remote measurements, it links patient-reported information to configurable clinical protocols. The platform combines adaptive questionnaires, medication reminders, rule-based alert generation, real-time messaging, and an exploratory artificial intelligence-assisted interaction module. This pilot study aimed to describe the system architecture, evaluate technical and operational feasibility during real-world deployment, characterize early usage patterns and monitoring signals, and assess perceived usability using the System Usability Scale (SUS). The pilot was not designed to establish clinical effectiveness or to validate the AI module as a diagnostic or therapeutic decision-support system.

**What is new?**
  - A novel contribution of this study is a disease-specific adaptive protocol engine, rather than a static questionnaire repository. This involves conditional symptom pathways that can change subsequent questions and trigger predefined clinical actions; medication support, structured monitoring, and clinician–patient messaging within a single workflow; a Progressive Web App architecture designed for cross-platform access and iterative hospital deployment; and an explicit separation between rule-based clinical monitoring and exploratory AI-assisted interaction.

## 2. Materials and Methods

### 2.1. Clinical Context and Target Population

**Figure 1:**
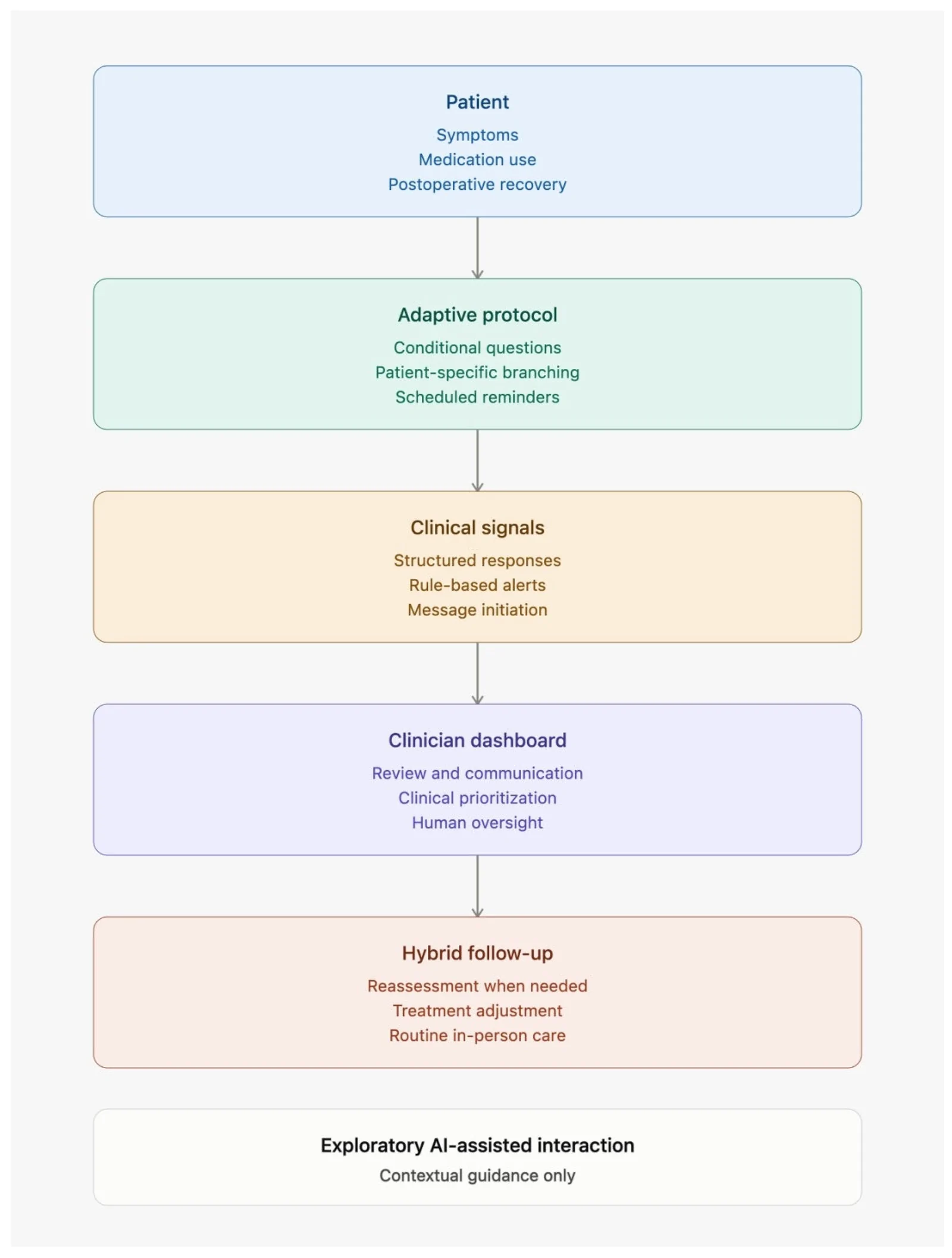
TeleGlaukos adaptive hybrid-care workflow. The workflow illustrates the progression from patient-reported symptoms, medication-related information, and recovery status through clinician-configurable adaptive protocols and structured clinical signals to clinician review and subsequent hybrid care. Exploratory AI-assisted interaction provides contextual guidance only, while clinically relevant outputs remain subject to ophthalmologist review.

TeleGlaukos was designed to support glaucoma patients requiring structured follow-up between face-to-face consultations, particularly in perioperative periods and during therapeutic surveillance. The intended clinical use includes postoperative glaucoma surgery follow-up, monitoring after treatment modification, symptom reporting between appointments, and medication adherence support. The platform was conceived as part of a hybrid care model in which remote monitoring complements, rather than replaces, conventional ophthalmology assessment [7] (Figure 1).

The target population includes postoperative glaucoma surgery patients, individuals undergoing medical treatment adjustments, and patients requiring continuous symptom reporting between appointments. Given the predominance of elderly users and patients with possible visual or cognitive limitations, particular emphasis was placed on accessibility throughout the platform design.

### 2.2. System Architecture

**Figure 2:**
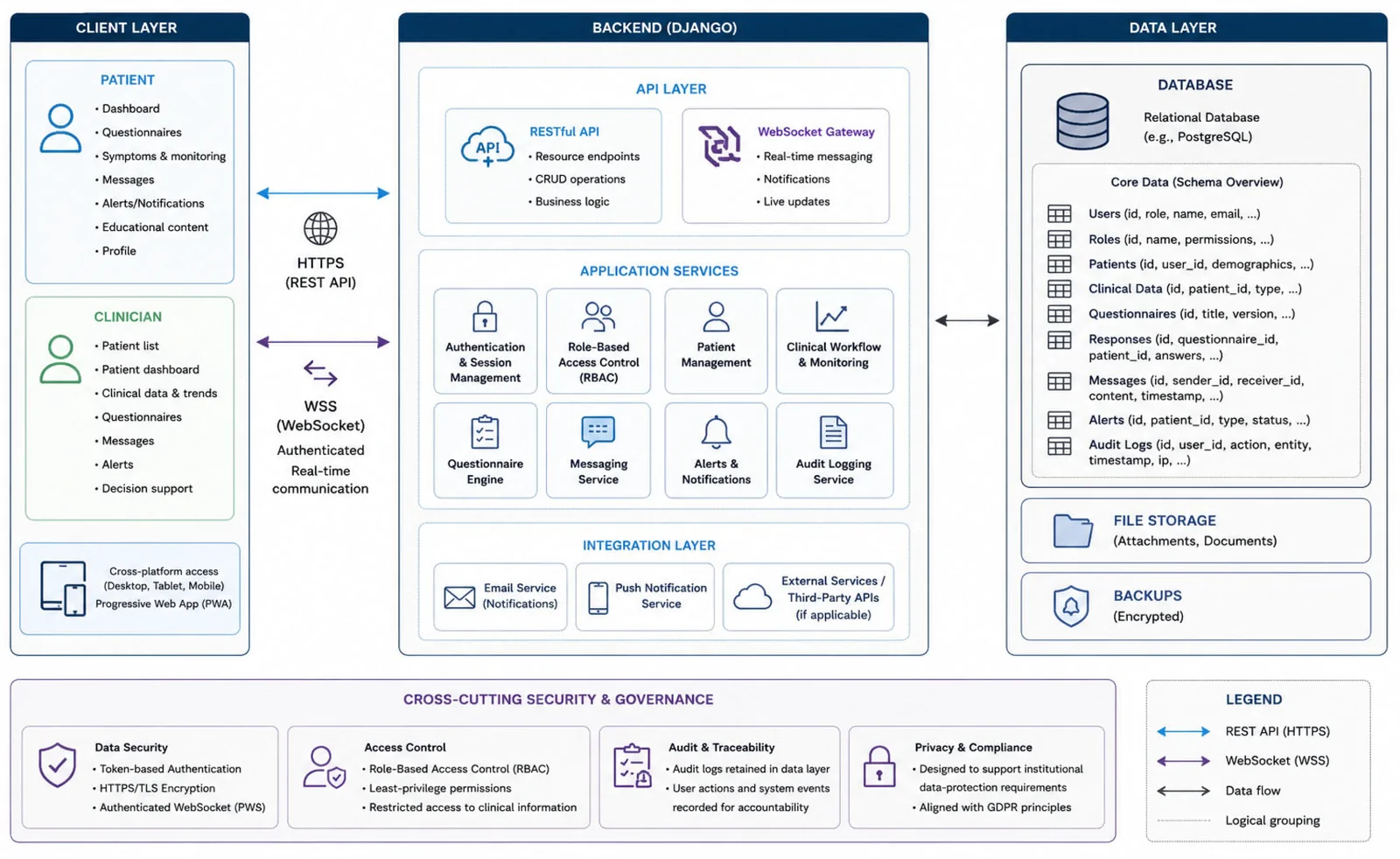
Technical architecture of the TeleGlaukos platform. The React Progressive Web App communicates with the Django backend through RESTful APIs and authenticated WebSocket connections. The backend comprises the protocol engine, application services, real-time communication layer, and associated data infrastructure. Patient and clinician interfaces are subject to role-based access control, while audit logs are retained within the data layer to support traceability.

TeleGlaukos adopts a modular, layered client-server architecture organized into a Progressive Web App (PWA) client, a Django-based backend, an asynchronous task-processing layer, and a data and infrastructure layer (Figure 2), with the main functional modules summarized in Table 1. This separation of presentation, business logic, real-time communication, and background processing supports maintainability, horizontal scalability, and incremental extension of monitoring functionality. The platform was deployed as a single modular Django application rather than as independently deployed microservices; the layers described here are logical modules within one deployable unit. The modular design also facilitates future interoperability with electronic health records using standards such as HL7 [10].

The client layer was implemented as a PWA using the React.js framework (version 18), with separate interfaces for patients and physicians. A PWA was selected instead of separate native applications to provide installation-like access across desktop, Android, and iOS devices while maintaining a single codebase. This approach reduces platform-specific development and maintenance, enables immediate deployment of updates without application-store review, and lowers the access barrier for users who may be reluctant or unable to install a native application. Responsive cross-platform access, offline caching through a service worker, and web push notifications support use in heterogeneous devices and connectivity conditions. These characteristics are particularly relevant to digital health tools intended for older or visually impaired populations, although accessibility must still be evaluated empirically [11]. SurveyJS is integrated on the frontend to render adaptive questionnaires and perform client-side conditional branching, while submitted responses are validated and persisted server-side. Questionnaire definitions and submitted responses are exchanged with the backend through RESTful APIs.

The backend was developed using Django and the Django REST Framework, providing token-based authentication, RESTful APIs, business logic, and relational data persistence. Real-time bidirectional communication is handled by Django Channels over WebSockets, supporting instant messaging, live clinician alerts, and notification propagation. Time-based and event-driven processes, including reminder scheduling, alert dispatch, questionnaire triggering, and notification delivery, are delegated to Celery workers and Celery Beat, with RabbitMQ as the message broker and Redis supporting the Channels layer and application caching. A relational database stores users, protocol definitions, questionnaire responses, messages, and audit logs (Figure 2).

Data security and privacy were addressed at the application level through token-based authentication, role-based access control with distinct patient and clinician permissions, and restricted access to clinical information. All client-server traffic, including REST and WebSocket connections, was transported over HTTPS/TLS, as required by the service-worker model of the Progressive Web App. Real-time communication was handled through authenticated WebSocket connections, and audit logs were retained within the data layer. The platform was designed in accordance with institutional data-protection requirements and the principles of the General Data Protection Regulation (GDPR).

### 2.3. Adaptive Protocol Engine and Medication Monitoring

The configurable protocol engine is the central innovation of TeleGlaukos. Protocols are composed of adaptive workflows (i.e., questionnaire pathways in which subsequent questions are determined by previous responses). Unlike static electronic forms, the questionnaire sequence can change according to previous responses, allowing the platform to omit irrelevant questions, request greater detail when a warning symptom is reported, and initiate a predefined alert or communication pathway. SurveyJS was integrated to support flexible adaptive form design and structured escalation pathways for postoperative complication screening, suspected disease progression, adverse drug reaction reporting, and adherence verification (Figure 1).

**Figure 3:**
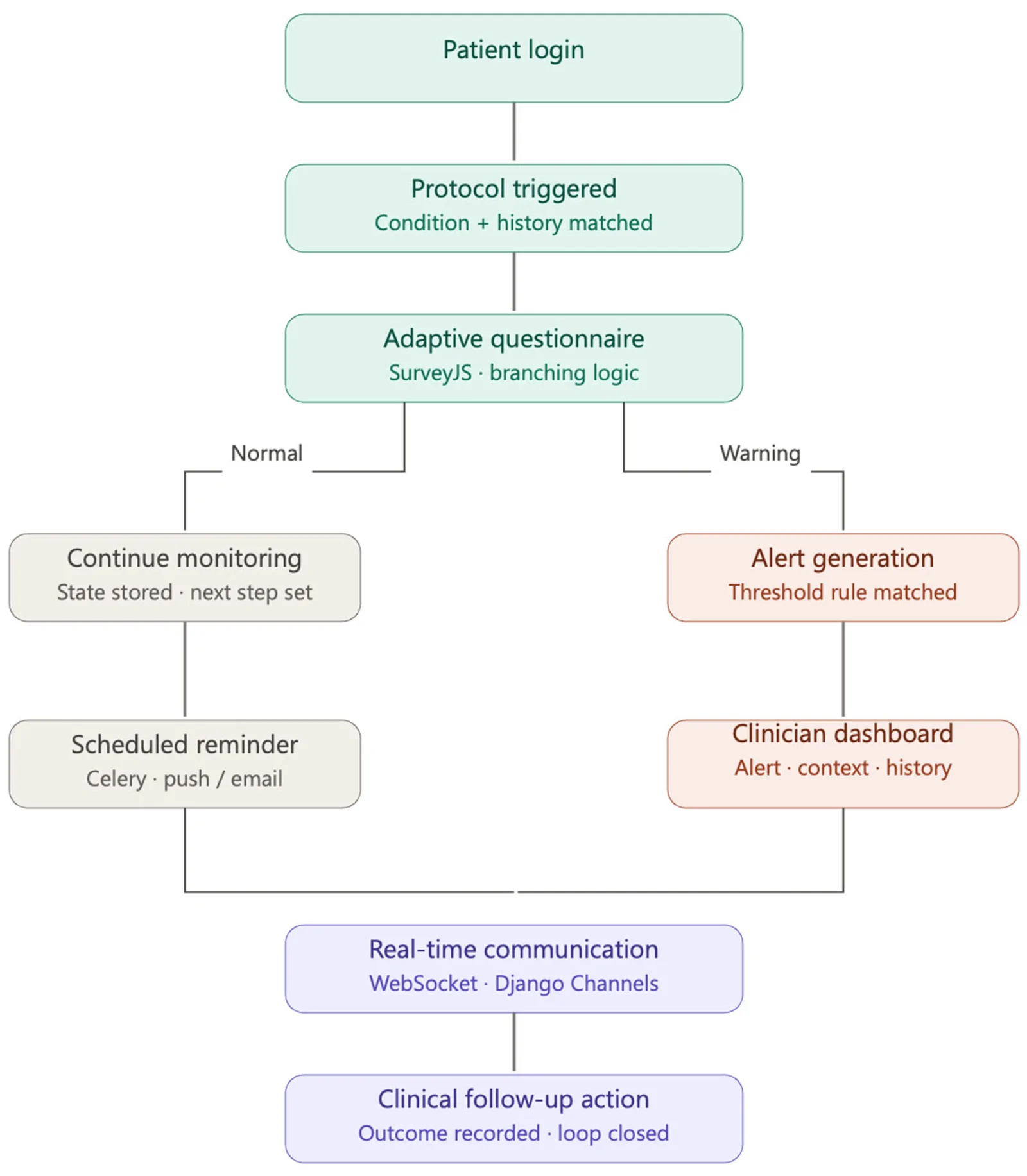
Adaptive telemonitoring workflow with symptom-based branching and clinician notification. Normal responses continue routine monitoring, whereas predefined warning criteria generate a clinician-facing alert and enable direct communication and, when indicated, clinical reassessment.

A typical workflow begins with a scheduled or symptom-initiated questionnaire. A negative response may close the workflow or schedule routine follow-up, whereas a positive response can reveal additional questions concerning onset, severity, laterality, associated symptoms, and medication exposure. The resulting structured information is then made available to the clinical interface and, where a clinician-defined rule is met, can generate an alert or prompt direct communication (rule-based strategy). The protocol engine therefore operationalizes clinical follow-up logic while preserving human review and allowing pathways to be modified without redesigning the full application (Figure 3).

The prescription subsystem enables digital prescription delivery, configurable reminder alarms, alert scheduling, and patient acknowledgement tracking. These mechanisms were designed to support early identification of adherence-related deviations and promote timely intervention when needed.

The alert rules were clinician-defined and based on predefined symptom pathways developed by glaucoma specialists, with warning criteria triggering clinician-facing alerts while routine responses remained within the standard monitoring pathway. They were intended to support structured clinical review and communication rather than to operate as validated diagnostic or predictive algorithms. Independent validation of alert sensitivity, specificity, and clinical performance was beyond the scope of this feasibility pilot.

All reports are submitted through idempotent API requests, in which an idempotency key derived from the patient identifier, report type, and reporting window ensures that repeated or concurrent submissions of the same input, irrespective of the originating channel, are resolved to a single stored record rather than creating a new one. The channels are furthermore functionally separated, with structured forms serving scheduled monitoring reports and the chat interface serving live assistance and ad hoc questions, so that duplicate clinical entries are avoided by design and the clinician’s review workload is unaffected.

Prior to fusion, all incoming inputs are normalized into a common structured representation: textual inputs are parsed directly, whereas documents uploaded in PDF form are processed by an OCR stage, with both the recognized text and the source file retained for traceability. The normalized records are retained in a relational database that acts as the single store for subsequent analysis, where deduplication at insertion time and aggregation of entries sharing the same patient and reporting window consolidate the raw inputs into one coherent clinical signal per episode.

### 2.4. Experimental AI-Assisted Functionalities

TeleGlaukos includes an exploratory AI-assisted module intended to facilitate contextual interaction within questionnaire workflows. It may provide conversational guidance or semantic response assistance, but it was not designed or used for autonomous diagnosis, risk stratification, alert prioritization, or independent therapeutic decision-making. Rule-based clinical protocols, rather than the large language model, generated the structured monitoring pathways described in this study, and all clinically relevant information remained subject to ophthalmologist review. This separation limits overstatement of the AI contribution and is consistent with current recommendations that ophthalmic AI should undergo task-specific evaluation, human oversight, privacy protection, bias assessment, and post-deployment governance before routine clinical use [12,13,14,15,16,17,18,19]. The module used a locally deployed Llama 3.2 large language model accessed through the server-side application layer. The model used only the conversational context and patient-provided questionnaire data available during the interaction. Inference was executed on-premises within the institutional infrastructure, on an Ubuntu 22.04.2 LTS machine with an NVIDIA GeForce RTX 3060 GPU (12 GB VRAM), an AMD Ryzen 9 5950X CPU, and 32 GB of RAM with 4 GB of swap. The same machine hosted the model both during development and during the pilot deployment, for consistency. Concurrent use of the assistant remained low throughout the pilot (a maximum of two simultaneous users), and this configuration was sufficient for interactive-latency responses at the observed request volume; deployments serving larger cohorts or time-critical interactions would require batched GPU serving and formal load testing to guarantee acceptable response times under peak concurrency.

During this pilot, AI-specific performance, frequency of use, response accuracy, user satisfaction, and clinical impact were not predefined endpoints and were therefore not analyzed separately. The present study consequently reports the AI component only as part of the platform architecture; formal validation will be required before any AI-mediated triage or decision-support function is considered for clinical implementation.

### 2.5. Pilot Deployment and Platform Analytics

**Figure 4:**
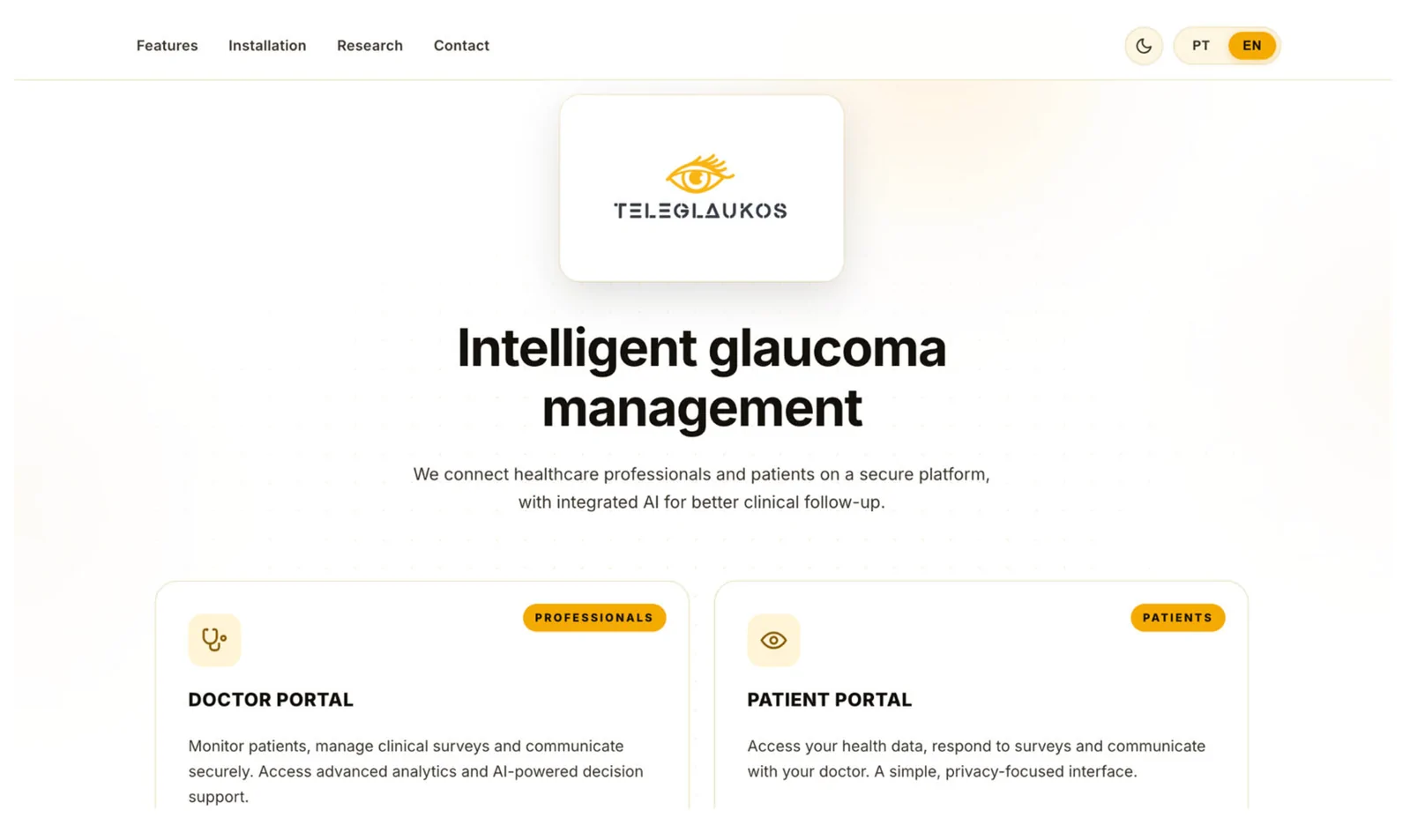
Public TeleGlaukos platform interface. The bilingual landing page provides separate access pathways for healthcare professionals and patients and presents the platform as a secure environment for clinical monitoring and communication.

**Figure 5:**
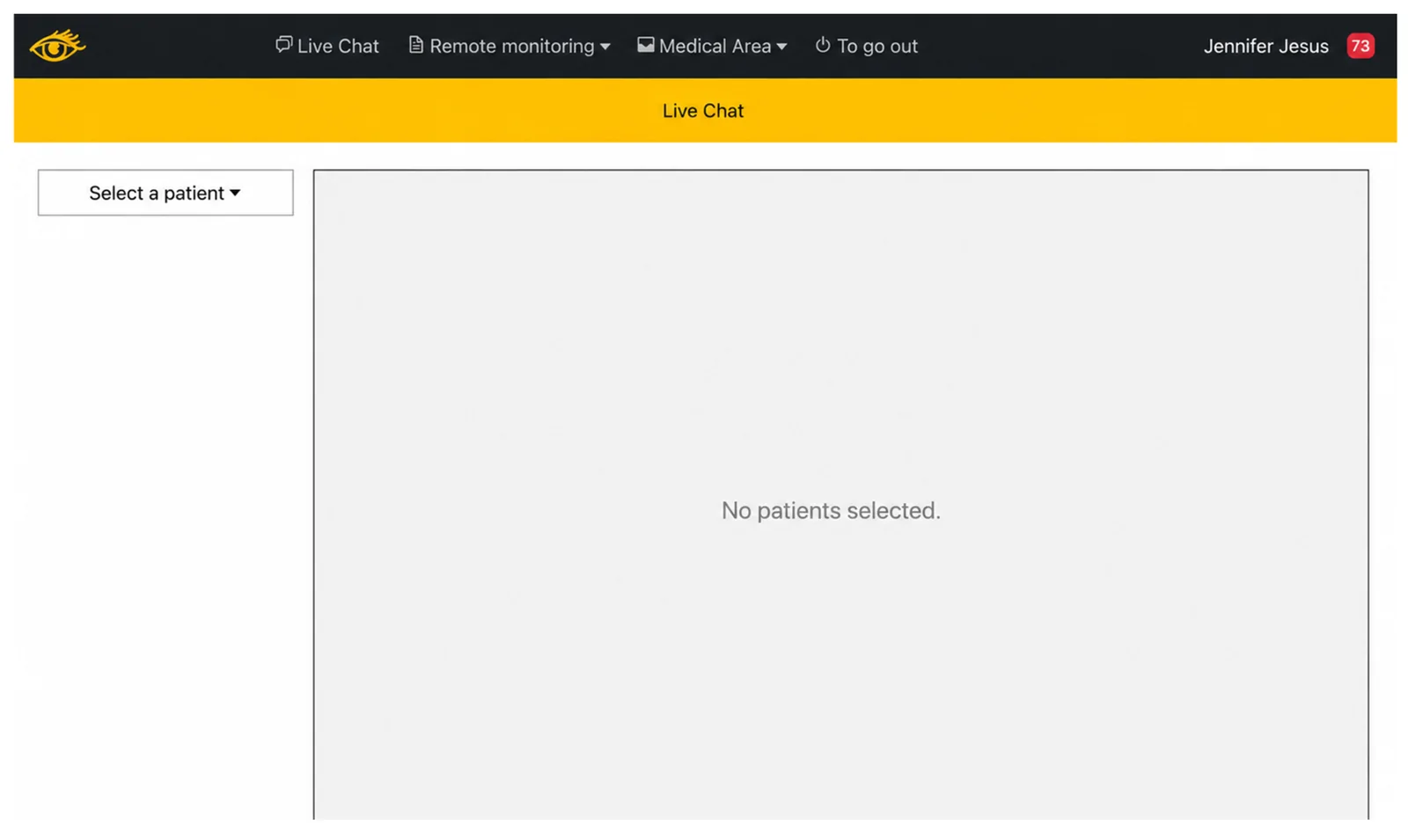
Clinician live-chat interface of the TeleGlaukos platform. The authenticated clinical portal enables the healthcare professional to select a patient using the left-side selector and access secure bidirectional communication, while the central panel indicates when no patient is selected.

This was a single-centre observational pilot study conducted at Unidade Local de Saúde Entre Douro e Vouga, Santa Maria da Feira. Thirty glaucoma patients under active follow-up at the glaucoma clinic were invited to participate after presentation of the project and provision of informed consent. These 30 patients constituted the clinical pilot cohort. The broader platform environment contained 95 registered user accounts: 30 glaucoma patients, 10 ophthalmologists, 10 research or administrative users, 20 development and testing accounts, and 25 users who registered but did not actively participate in the clinical pilot (Table 2). Only the 30 enrolled glaucoma patients were included in the clinical pilot analyses. Ophthalmologists involved in the project could access the platform in their clinical role, and four completed the usability assessment. The deployment ran from May 2025 to April 2026, during which enrolled patients used TeleGlaukos for postoperative and therapeutic follow-up alongside usual outpatient care. The primary outcome of the pilot was perceived usability, assessed using the SUS. Secondary exploratory outcomes included platform usage metrics, questionnaire completion, communication activity, and clinically relevant signals identified through structured monitoring (Figure 4 and Figure 5).

Electronic informed consent was recorded before enrolment and use of the TeleGlaukos monitoring workflows. The consent process covered participation in the pilot and the use of study data for research purposes. Participation was voluntary, and withdrawal from the study did not affect standard clinical care. The platform was not intended to replace urgent ophthalmic assessment, and patients were instructed to use standard clinical pathways for urgent symptoms.

Platform usage was recorded using Google Analytics 4 (GA4; Google LLC, Mountain View, CA, USA). GA4 was selected because its event-based data model provided standardized, reproducible measures of navigation and interaction without requiring a separate analytics infrastructure during this feasibility deployment. Engaged sessions were defined according to the platform’s standard metric as sessions lasting longer than ten seconds, containing a conversion event, or comprising at least two page or screen views; events represented individual user interactions logged by the analytics platform. Analytics were interpreted at platform level and could therefore include activity generated by clinical users, non-pilot registered users, and development or testing accounts. Measures of registered accounts, engaged sessions, recorded events, questionnaire submissions, and clinician–patient messaging were extracted. Clinical signals and SUS analyses were restricted to the relevant pilot participants. GA4 was used to characterize overall platform activity rather than individual patient behaviour, clinical effectiveness, adherence, or patient-level exposure. Perceived usability was evaluated among patients who had used the application for several weeks, together with participating ophthalmologists. Data were analyzed descriptively.

### 2.6. System Usability Scale Evaluation

**Figure 6:**
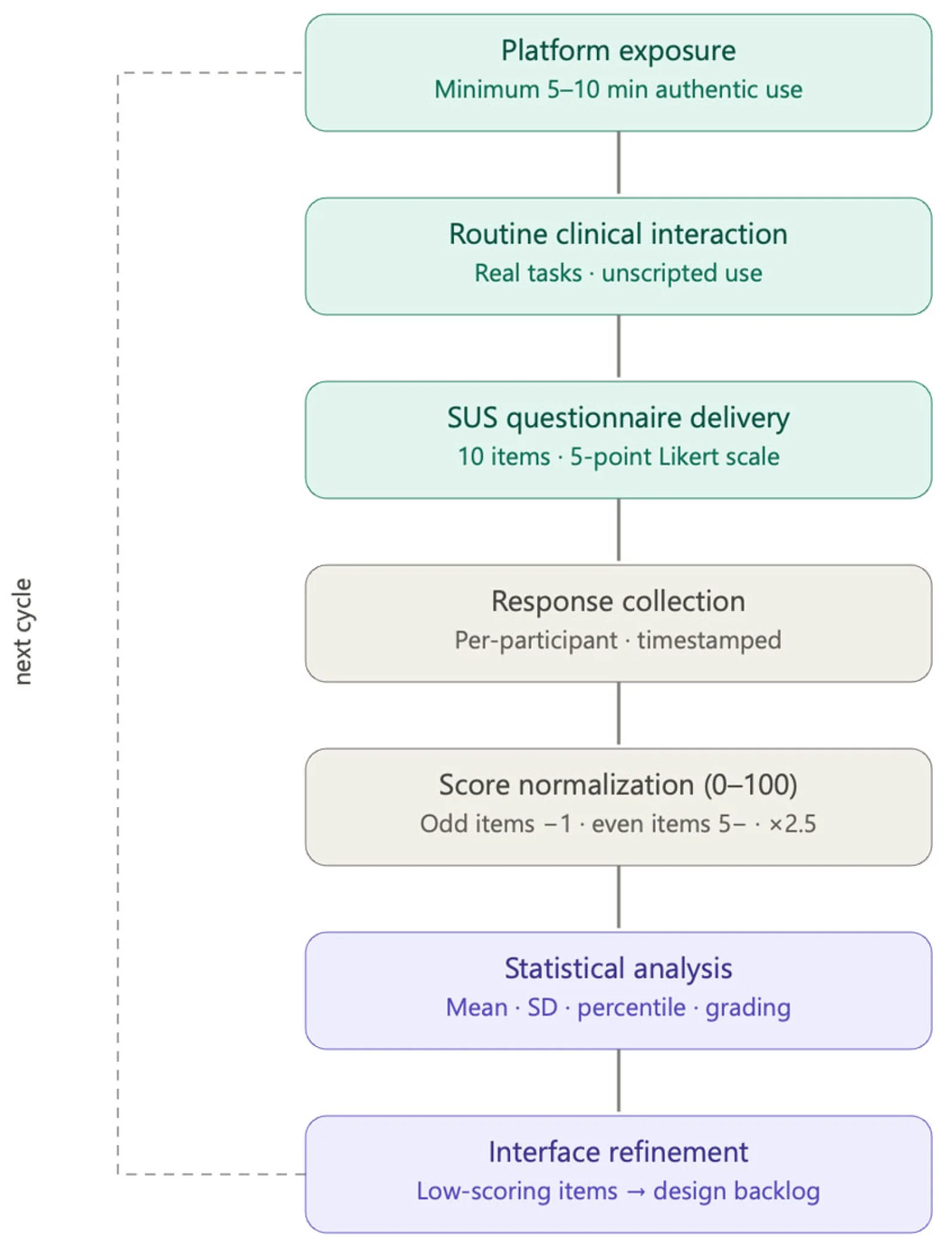
SUS evaluation workflow used in the TeleGlaukos pilot, from platform exposure and questionnaire delivery to scoring, statistical analysis, and interface refinement.

**Figure 7:**
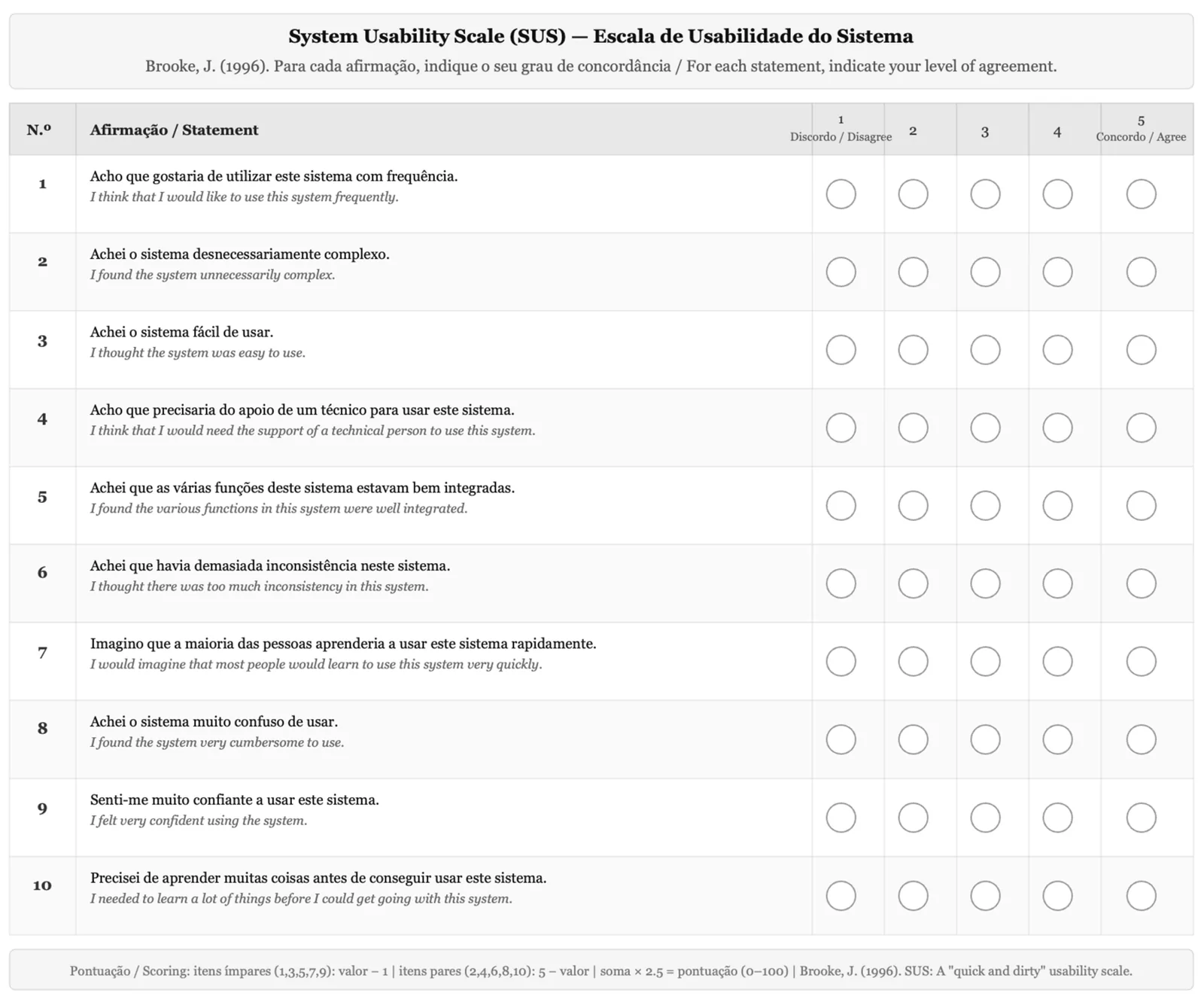
Portuguese version of the System Usability Scale questionnaire administered to participating patients and ophthalmologists, based on the original SUS developed by Brooke [20].

Perceived usability was assessed using the SUS [20], a ten-item Likert-based instrument widely used to evaluate interactive systems. The questionnaire was administered in Portuguese through an online form (Google Forms, Google LLC, Mountain View, CA, USA) to participating patients and ophthalmologists. Each item is rated on a five-point scale. Scores were calculated according to standard SUS methodology: for positively worded items, one point was subtracted from the response; for negatively worded items, the response was subtracted from five; the resulting sum was multiplied by 2.5 to produce a normalized score from 0 to 100. Overall scores were interpreted using established adjective and acceptability benchmarks [21,22]. Evidence from digital health applications supports the use of SUS as a concise benchmark for perceived usability, although usability alone does not establish clinical effectiveness or implementation success [23,24] (Figure 6 and Figure 7).

### 2.7. Statistical Analysis

Continuous variables are reported as mean ± standard deviation (SD), median, range, and 95% confidence intervals (CIs) when appropriate. Internal consistency of the SUS instrument was assessed using Cronbach’s alpha. All SUS items were configured as mandatory, ensuring complete item-level data for submitted questionnaires. Participants who did not initially complete the SUS were contacted and encouraged to respond. Because of the small ophthalmologist subgroup, SUS scores between patients and ophthalmologists were compared using the Mann–Whitney U test. Effect size was reported using rank-biserial correlation and Cohen’s d. Statistical significance was defined as *p* < 0.05. Statistical analyses were performed using IBM SPSS Statistics, version 30 (IBM Corp., Armonk, NY, USA).

## 3. Results

### 3.1. Platform Deployment and Usage

The platform remained operational throughout the pilot period and supported remote follow-up without technical failures requiring suspension of deployment. A total of 95 user accounts were registered: 30 glaucoma patients, 10 ophthalmologists, 10 research or administrative users, 20 development and testing accounts, and 25 non-pilot users who registered but did not actively participate in the clinical pilot. Only the 30 glaucoma patients constituted the clinical pilot cohort. Across the full platform environment, 737 engaged sessions and 1329 recorded events were logged. Platform access occurred through desktop and mobile devices. Thirteen pilot patients actively used the communication module, generating 30 bidirectional messages between patients and physicians. Communication modules showed the highest engagement, with mean session durations of 4 min 14 s in the patient chat and 3 min 13 s in the physician chat. Questionnaire-based monitoring generated 186 completed responses. Because analytics included the broader registered environment, these platform-level metrics should not be interpreted as patient-only utilization measures (Figure 5). The demographic and clinical characteristics of the 30-patient pilot cohort are summarized in Table 3 and pilot deployment and platform usage characteristics are summarized in Table 4.

Clinician response time to patient messages was not predefined as an outcome and was therefore not analyzed in this pilot. Future prospective evaluations should record message timestamps and report median response time, out-of-hours activity, unresolved messages, and the proportion of contacts leading to clinical reassessment.

### 3.2. Clinical Monitoring Signals

Questionnaire-based monitoring identified suspected treatment-related adverse reactions in two patients after initiation of new topical therapy. Both patients reported periocular irritation through the structured remote pathway. These symptoms were captured through the questionnaire pathway and flagged within the clinician interface for review by the physician responsible for the patient’s follow-up. These reports were then visible to the clinical team between scheduled appointments and led to targeted reassessment followed by treatment adjustment. The cases are clinically relevant because ocular-surface and periocular adverse effects can reduce persistence with topical therapy and may otherwise lead patients to discontinue treatment without supervision. Although the pilot was not designed to determine diagnostic accuracy, prevent urgent visits, or prove a causal effect on outcomes, these cases provide a concrete feasibility signal: adaptive symptom reporting can convert an otherwise unstructured complaint into an actionable clinical review pathway.

### 3.3. Exploratory AI Component

The AI-assisted interaction module was available as an architectural component, but AI-specific usage frequency, response quality, safety, user satisfaction, and clinical impact were not predefined outcomes. No clinical alert, reassessment, or treatment adjustment reported in this pilot was attributed to autonomous AI decision-making. Accordingly, no effectiveness claim is made for the AI module, and its contribution should be interpreted as exploratory functionality requiring a separate validation study.

### 3.4. System Usability Scale Results

**Figure 8:**
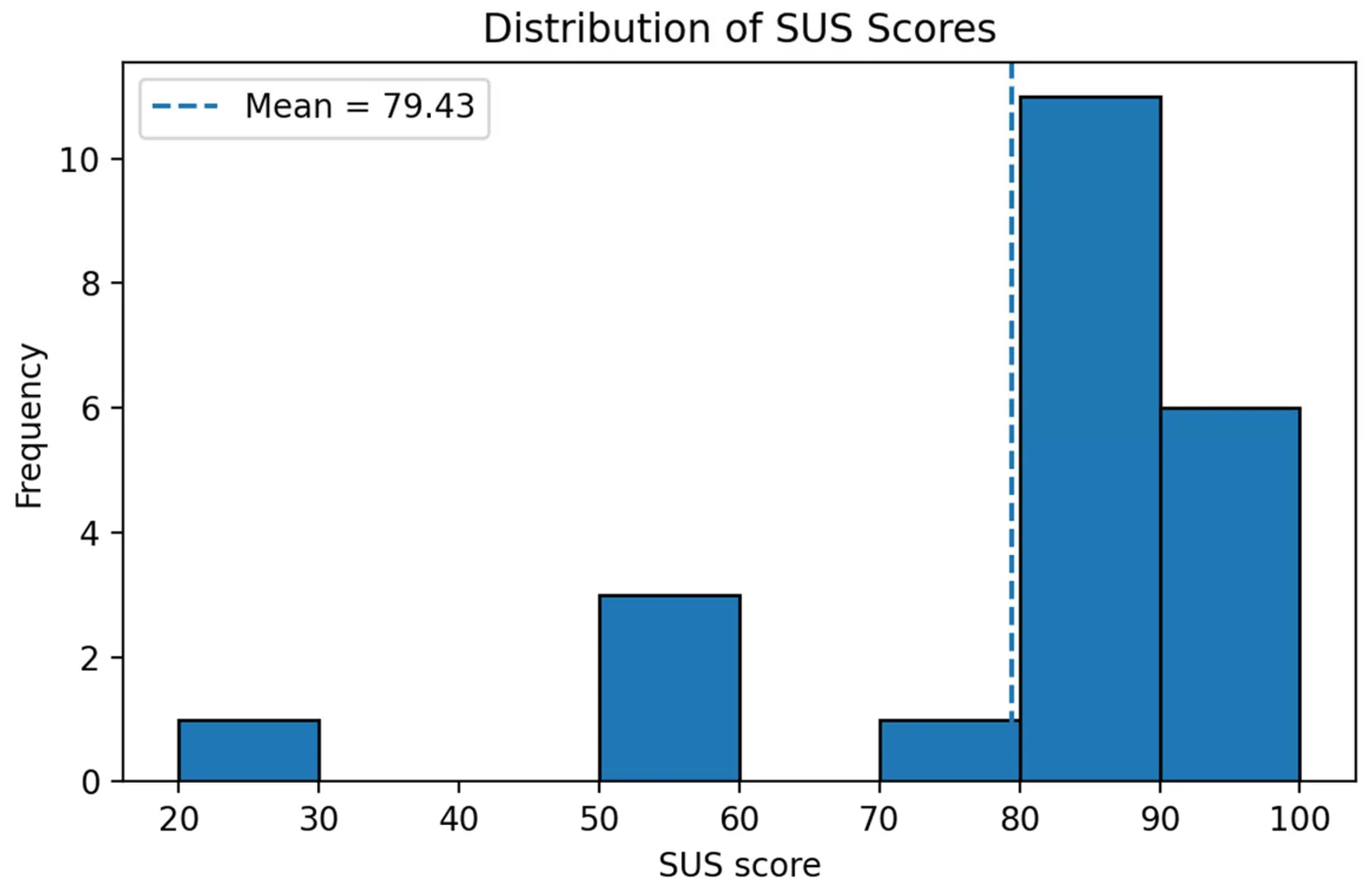
Distribution of overall System Usability Scale scores. The dashed line indicates the mean SUS score (79.43).

**Figure 9:**
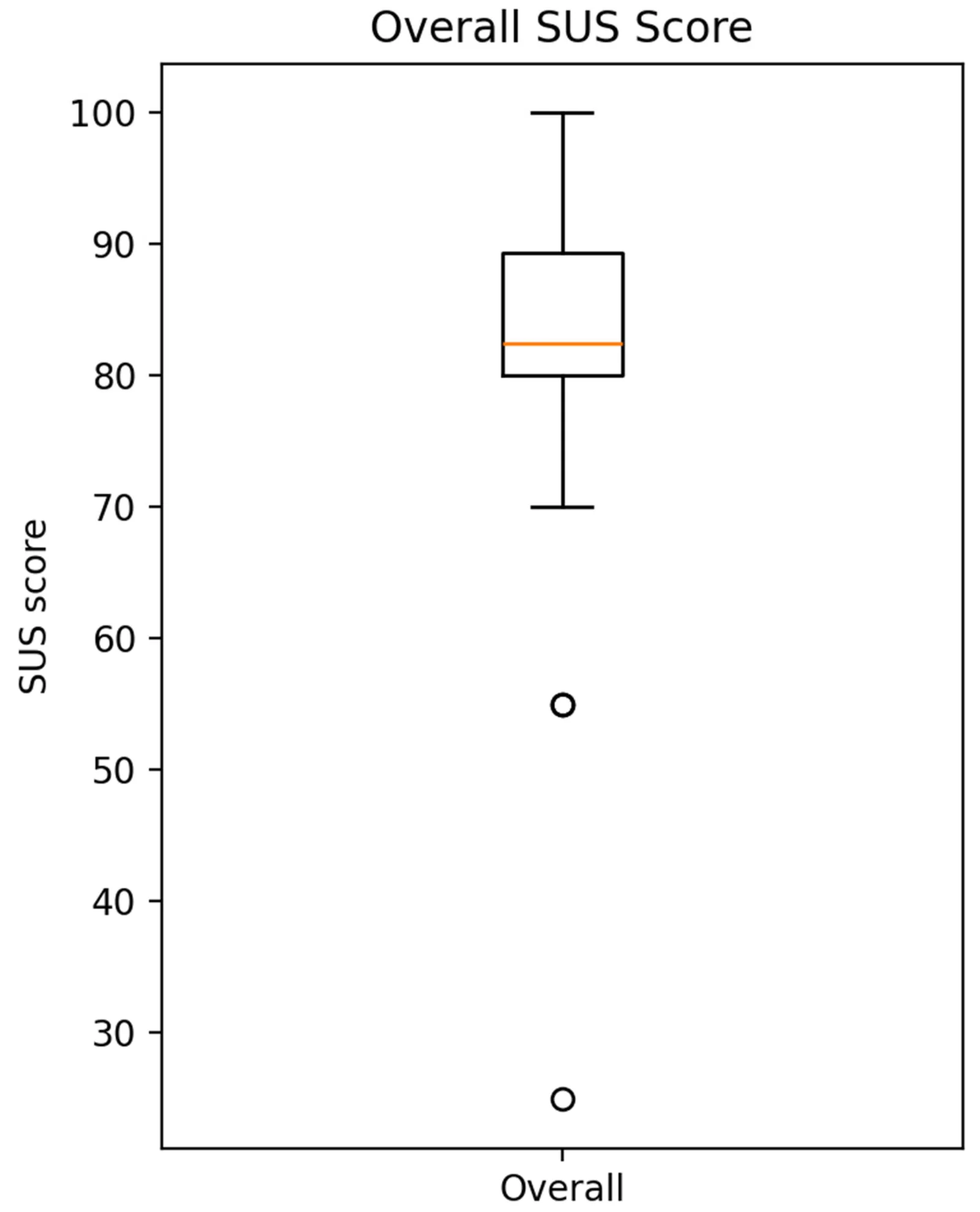
Overall System Usability Scale score boxplot.

A total of 22 participants completed the SUS questionnaire, including 18 patients and four ophthalmologists. The overall mean SUS score was 79.43 ± 17.98 (95% CI: 71.46–87.40), with a median of 82.50 and a range from 25 to 100. The questionnaire demonstrated high internal consistency, with Cronbach’s alpha = 0.882. Additional usability outcomes are summarized in Table 5 (Figure 8 and Figure 9).

**Figure 10:**
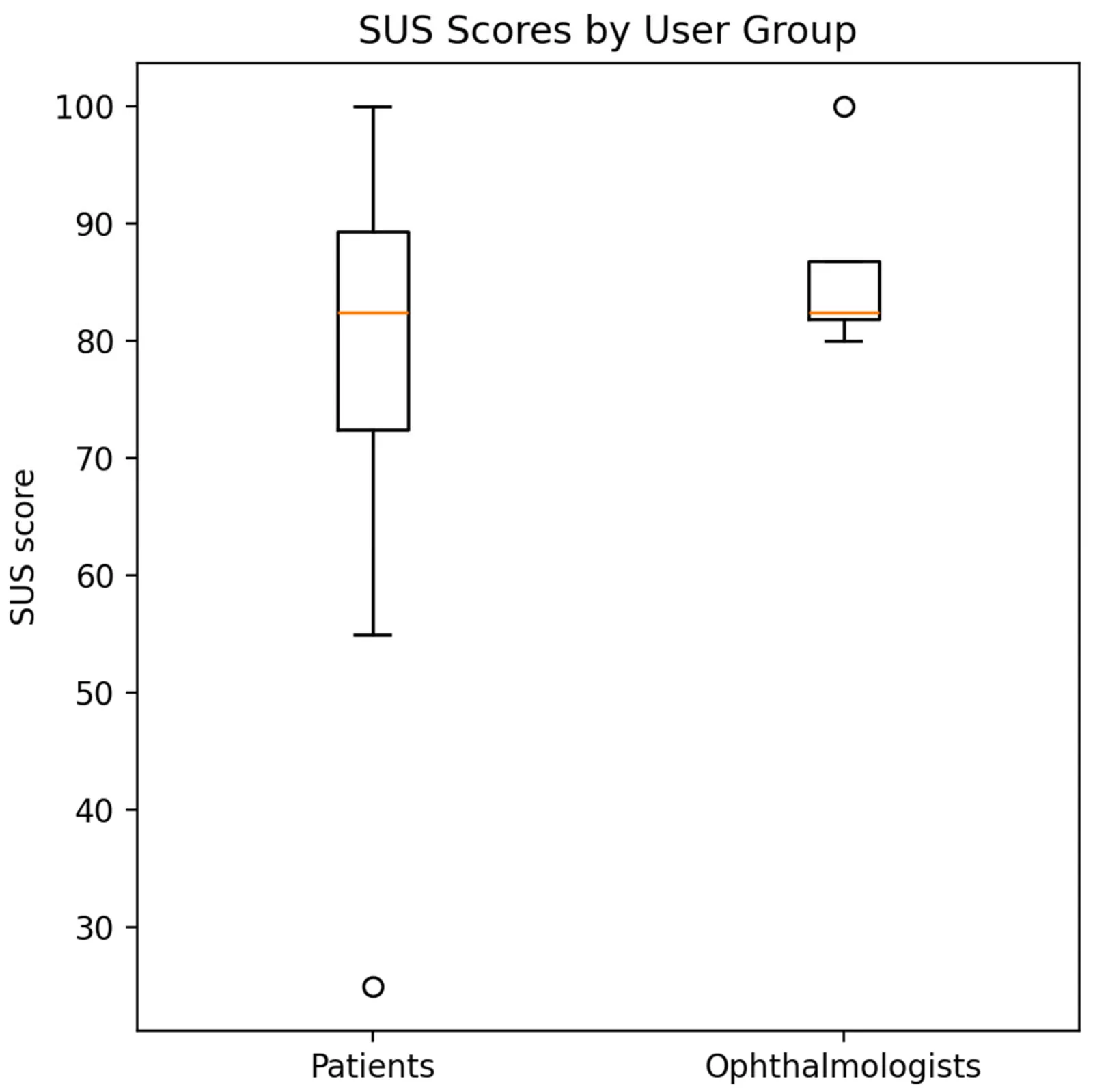
Comparison of System Usability Scale scores between patients and ophthalmologists.

**Figure 11:**
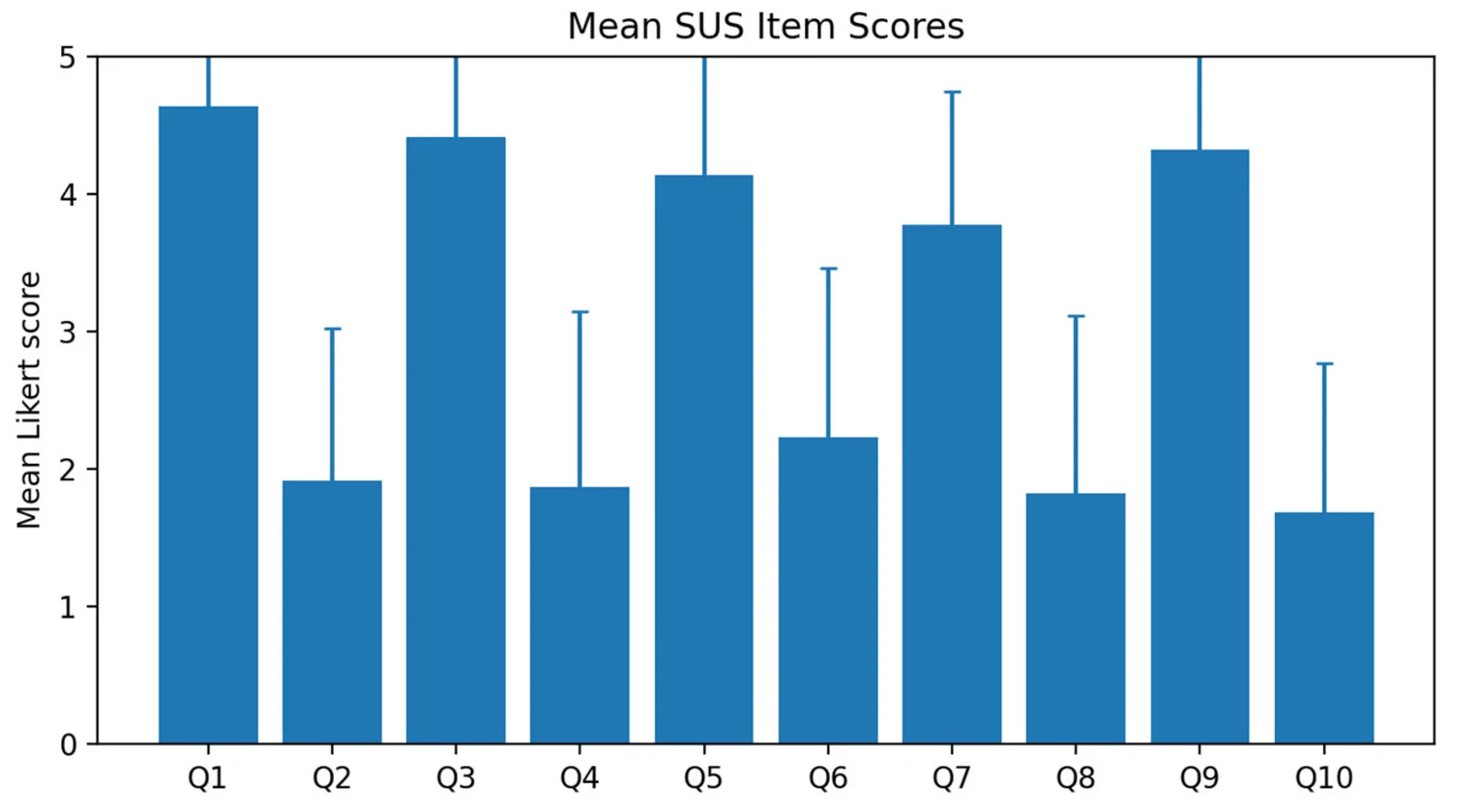
Mean Likert score for each System Usability Scale item. Error bars represent standard deviations.

According to established SUS benchmark interpretations, the observed score corresponds to a Good adjective rating and falls within the Acceptable usability range. Patients achieved a mean SUS score of 77.92 ± 19.26, whereas ophthalmologists achieved 86.25 ± 9.24. Although ophthalmologists rated the platform more positively, the difference was not statistically significant (Mann–Whitney U = 40.5, *p* = 0.731), with a small effect size (rank-biserial correlation = 0.125) (Figure 10). At the item level, agreement was highest for intention to use the platform frequently (Q1, mean 4.64) and confidence in using the system (Q9, mean 4.32). Negatively worded items received low scores; notably these included, perceived need to learn a great deal before using the system (Q10, mean 1.68) and system complexity (Q2, mean 1.91) (Table 6) (Figure 11).

## 4. Discussion

TeleGlaukos addresses a gap between measurement-centred teleglaucoma and the everyday need to manage symptoms, medication use, and communication between scheduled visits. Reported telemonitoring approaches in glaucoma have largely emphasized home tonometry, remote perimetry, imaging, or virtual specialist assessment [6,9] (Table 7). TeleGlaukos adds a complementary layer: a clinician-configurable protocol engine that transforms patient-reported information into adaptive questions, structured signals, predefined escalation pathways, and bidirectional communication. Previous work has demonstrated the feasibility of web-based symptom self-monitoring in glaucoma, while chatbot-assisted postoperative symptom reporting with bidirectional clinical feedback has also been explored in other ophthalmic settings, including after intravitreal injections [25,26]. TeleGlaukos builds on these concepts by combining adaptive symptom reporting with an integrated communication channel, allowing the clinical team to clarify reported symptoms or concerns when necessary. The pilot demonstrated that this architecture could operate in a real-world clinical setting without requiring installation of separate native applications.

Platform analytics suggest early interaction with both monitoring and communication components, as reflected by 186 questionnaire responses and 30 bidirectional messages. However, the distinction between 95 registered accounts and the 30-patient clinical cohort is essential: platform-level analytics included clinicians, research or administrative users, development and testing accounts, and non-pilot registered users. The session and event totals should therefore be interpreted as evidence of technical and operational activity rather than as direct measures of patient adherence or clinical exposure. The most clinically informative observation was the identification of two suspected treatment-related adverse reactions. In both cases, structured symptom reporting brought a problem to clinical attention before the next routine appointment and enabled reassessment and treatment adjustment. These cases do not establish effectiveness, but they illustrate the mechanism by which adaptive telemonitoring may reduce the period during which adverse effects remain unreported, support supervised treatment changes, and strengthen continuity within hybrid care pathways [7,9,27].

The adaptive questionnaire design is central to this mechanism. Static forms collect the same information from every user, whereas conditional workflows can concentrate on clinically relevant details and reduce unnecessary questions. For glaucoma follow-up, this allows a protocol to distinguish routine recovery from potentially concerning symptoms and to request additional contextual information before clinician review. The principal engineering contribution is therefore not the digitization of a questionnaire alone, but the separation of questionnaire content, branching logic, alert rules, and communication functions into configurable components that can be reused across perioperative and therapeutic pathways.

The usability analysis supports the user-centred design of TeleGlaukos. The overall SUS score of 79.43 corresponds to a good adjective rating and an Acceptable usability classification, with high internal consistency (Cronbach’s alpha = 0.882). This score exceeds the commonly cited SUS benchmark of approximately 68 and is favourable in comparison with pooled estimates reported for digital health applications [23]. The absence of a statistically significant difference between patients and ophthalmologists suggests broadly comparable perceived usability, although the small ophthalmologist subgroup limits inference. Evidence from telehealth research indicates that ease of use, perceived usefulness, digital literacy, training, and connectivity strongly influence adoption [24,28,29,30,31,32]. These factors are particularly relevant in glaucoma populations, which often include older adults with visual impairment and variable familiarity with digital tools.

The modular architecture is an additional translational strength. Separating the PWA interface, protocol logic, asynchronous task processing, real-time communication, and data persistence allows individual components to be updated without redesigning the entire system. This approach may facilitate scalability, deployment across different clinical pathways, and future integration with institutional information systems. Interoperability through HL7 FHIR could support structured exchange of patient, medication, questionnaire, and clinical-event data, although this functionality was not implemented or evaluated in the present pilot [10]. From an implementation perspective, successful adoption will also require defined clinical responsibilities, response-time expectations, data governance, cybersecurity safeguards, and dedicated professional time for reviewing alerts and messages.

### Clinical Implications

Adaptive symptom pathways may make clinically relevant complaints visible between scheduled visits. Structured reporting can support targeted reassessment and supervised treatment adjustment. A single cross-platform interface may simplify access for patients and clinicians. Platform-level engagement metrics must be separated from patient-level clinical outcomes. AI-assisted interaction should remain subordinate to validated protocols and clinician oversight.

Several limitations should be acknowledged. First, this was a single-centre observational pilot without a randomized control group or direct comparison with standard follow-up; clinical effectiveness, reduction in urgent visits, adherence improvement, cost-effectiveness, and long-term disease stability cannot therefore be inferred. Second, the clinical cohort and SUS sample were small, particularly the ophthalmologist subgroup, limiting statistical power and generalizability. Moreover, although the involvement of relatives or caregivers was encouraged when needed to support patients with limited digital literacy, selection bias remains possible, as patients who were willing to engage with a web-based platform may have greater digital literacy, motivation, or engagement than the broader glaucoma population. Third, Google Analytics described activity across the broader platform environment rather than patient-only exposure; development accounts and non-pilot users may have contributed to session and event counts. Fourth, analytics do not establish whether an interaction was clinically meaningful, whether reminders improved medication adherence, or whether questionnaires were completed independently. Family assisted completion may have influenced both usability ratings and self-reported data. Fifth, the two suspected adverse reactions represent feasibility signals rather than validated diagnostic outcomes. Finally, the AI-assisted component was not evaluated through predefined performance, safety, usability, or clinical-impact endpoints. It should therefore be regarded as an experimental architectural feature, not as validated clinical decision support. Any future clinical use will require prospective validation, human oversight, privacy and security safeguards, bias assessment, transparent failure handling, and formal governance [12,13,14,15].

Future work should include prospective multicentre validation, longer follow-up, and comparison with standard outpatient monitoring. Relevant outcomes include medication adherence, time to clinical reassessment, urgent care utilization, safety events, patient and caregiver burden, clinician workload, satisfaction, and cost-effectiveness. Patient-level analytics should be separated from development and administrative activity, with predefined engagement metrics and clear denominators. Accessibility evaluation should include older adults with different levels of visual function and digital literacy, using interviews, think-aloud testing, and caregiver-assisted use analyses. Further development should prioritize electronic health record interoperability through FHIR-based exchange and a formally specified clinical workflow for alerts. The AI-assisted module should be evaluated separately using predefined tasks, expert-reviewed reference standards, accuracy and safety metrics, subgroup analyses, privacy testing, and prospective human-in-the-loop validation before any triage or recommendation function is deployed.

The platform architecture was intentionally designed around configurable protocols and modular services that could support deployment across multiple clinical sites. Nevertheless, multicentre scalability has not yet been demonstrated and will require site-specific governance, harmonized clinical pathways, interoperable data standards, and prospective evaluation across different healthcare settings.

## 5. Conclusions

TeleGlaukos provides preliminary evidence of the technical feasibility and favourable perceived usability of a clinician-led adaptive telemonitoring platform for hybrid perioperative and therapeutic glaucoma care. Its principal contribution is a configurable protocol engine that links conditional questionnaires, medication-related monitoring, structured alerts, and bidirectional communication rather than relying on static forms or remote measurements alone. Within the 30-patient clinical pilot, structured symptom reporting identified two suspected treatment-related adverse reactions and prompted clinical reassessment and treatment adjustment. The wider platform contained 95 registered accounts; platform-level analytics must therefore remain distinct from patient-only outcomes. The Progressive Web App architecture supports cross-platform deployment and iterative development, while the AI-assisted component remains exploratory and unvalidated. Larger prospective multicentre studies should evaluate patient-level engagement, safety, effectiveness, adherence, clinician workload, interoperability, and cost-effectiveness before routine implementation.

## Figures and Tables

**Table 1:** Functional modules of TeleGlaukos.

Module	Primary Function	Technology Used	Clinical Relevance
Authentication layer	Secure role-based access	Django Auth and SSO integration	Privacy and controlled access
Adaptive protocol engine	Dynamic questionnaires and branching logic	SurveyJS + custom logic in the backend	Personalized follow-up
Real-time messaging	Bidirectional communication	Django Channels/WebSockets	Immediate intervention and continuity
Prescription management	Medication scheduling and reminders	Django scheduler with Celery	Therapeutic adherence support
Notification system	Event-triggered alerts	WebSocket push/Service Workers	Early warning escalation
Clinical record viewer	Access to patient documentation	Django ORM/API	Longitudinal tracking
AI clinical assistant	Contextual semantic assistance	LLM integration layer using local models (llama3.2)	Experimental workflow augmentation

**Table 2:** Composition of the registered platform environment and clinical pilot cohort.

User Category	*n*	Role in the Study
Glaucoma patients	30	Clinical pilot cohort
Ophthalmologists	10	Clinical users; 4 completed SUS
Research/administrative users	10	Platform support and project administration
Development/testing accounts	20	Software development and quality testing
Registered non-pilot users	25	Created an account but did not actively participate in the clinical pilot
Total registered accounts	95	Platform environment

**Table 3:** Demographic and clinical characteristics of the clinical pilot cohort (*n* = 30).

Characteristic	Pilot Cohort (*n* = 30)
Age, mean ± SD (years)	64.2 ± 15.0
Female sex, *n* (%)	14 (46.7%)
Male sex, *n* (%)	16 (53.3%)
Primary open-angle glaucoma, *n* (%)	20 (66,7%)
Other glaucoma types, *n* (%)	10 (33.3%)
Mild disease, *n* (%)	8 (26.7%)
Moderate disease, *n* (%)	12 (40.0%)
Advanced disease, *n* (%)	10 (33.3%)
Medical treatment only, *n* (%)	15 (50.0%)
Previous/following glaucoma surgery, *n* (%)	15 (50.0%)

**Table 4:** Pilot deployment and platform usage characteristics.

Parameter	Value
Pilot population	30 glaucoma patients
Total registered platform accounts	95
Engaged sessions	737
Recorded events	1329
Completed questionnaire responses	186
Patients using communication module	13
Bidirectional messages	30
Mean patient chat session duration	4 min 14 s
Mean physician chat session duration	3 min 13 s
Real-time communication	Enabled
Medication adherence reminders	Enabled
Controlled comparison arm	Not included

**Table 5:** Summary of SUS outcomes and benchmark interpretation.

Metric	Value
Participants completing SUS	22
Patients	18
Ophthalmologists	4
Overall SUS score	79.43 ± 17.98
95% CI	71.46–87.40
Median (range)	82.50 (25–100)
Cronbach’s alpha	0.882
SUS adjective rating	Good
Acceptability range	Acceptable
Mann–Whitney U	40.5
*p* value	0.731
Rank-biserial correlation	0.125
Cohen’s d	0.46

**Table 6:** SUS item-level descriptive statistics.

Item	Short Statement	Mean	SD
Q1	Use frequently	4.64	0.58
Q2	Unnecessarily complex	1.91	1.11
Q3	Easy to use	4.41	0.80
Q4	Need technical support	1.86	1.28
Q5	Functions well integrated	4.14	0.89
Q6	Too much inconsistency	2.23	1.23
Q7	Most people would learn quickly	3.77	0.97
Q8	Cumbersome/confusing to use	1.82	1.30
Q9	Felt confident using the system	4.32	0.84
Q10	Needed to learn many things first	1.68	1.09

**Table 7:** Conceptual comparison of the principal emphasis of reported teleglaucoma approaches and the workflow contribution of TeleGlaukos. This synthesis is based on the literature review and recent overview of telemedicine in glaucoma [6,9], rather than a head-to-head evaluation.

Dimension	Common Emphasis in Reported Teleglaucoma Approaches	TeleGlaukos Contribution
Primary data	Remote IOP, visual field, imaging, or virtual consultation	Symptoms, medication use, postoperative recovery, and communication
Questionnaire logic	Often fixed or assessment-specific	Conditional, patient-specific branching with clinician-defined rules
Clinical workflow	Remote measurement followed by review	Structured escalation, alerts, messaging, and hybrid reassessment
AI role	Variable across solutions	Exploratory interaction only; no autonomous clinical decision-making

## Data Availability

The data presented in this study are available on request from the corresponding author. The data are not publicly available due to ethical and privacy restrictions.

## References

[1] Wang Z., Xue C.C., Li Y., Wu Y., Pan Z., Li F., Cheung C.Y., Ohno-Matsui K., Friedman D.S., Garway-Heath D. (2026). Global Glaucoma Prevalence: Burden and Projection to 2060. Am. J. Ophthalmol..

[2] Bhartiya S., Ichhpujani P., Wadhwani M. (2025). Current Perspectives in Tackling Glaucoma Blindness. Indian J. Ophthalmol..

[3] Inayat H., Abdul-Nabi M., Leung B., Jiang J., Robertson S., Malvankar-Mehta M.S. (2024). Detrimental Impact on Work Productivity in Patients with Glaucoma: A Systematic Review. JFO Open Ophthalmol..

[4] Stagg B.C., Granger A., Guetterman T.C., Hess R., Lee P.P. (2022). The Burden of Caring for and Treating Glaucoma: The Patient Perspective. Ophthalmol. Glaucoma.

[5] Cardoso-Teixeira P., Aguiar C.P., Ambrósio J.A., Pedro J.C., Figueiredo L. (2025). Managing Outpatient Consultations in Ophthalmology: Analyzing First Consultations Outcomes. Rev. Soc. Port. Oftalmol..

[6] Jesus J., Aguiar C., Meira D., Rodriguez-Una I., Beirão J.M. (2025). Telemonitoring Tools for Glaucoma Patients: A Systematic Review of Current Trends and Applications. J. Clin. Med..

[7] Toschi E., Adam A., Frimpong N., Hurlbert R., Slyne C., Laffel L., Munshi M. (2024). Hybrid Care Model: Combining Telemedicine and Office Visits for Diabetes Management in Older Adults with Type 1 Diabetes. Med. Res. Arch..

[8] Anderson A.J., Bedggood P.A., Kong Y.X.G., Martin K.R., Vingrys A.J. (2017). Can Home Monitoring Allow Earlier Detection of Rapid Visual Field Progression in Glaucoma?. Ophthalmology.

[9] Jo J.J., Pasquale L.R. (2024). Recent Developments of Telemedicine in Glaucoma. Curr. Opin. Ophthalmol..

[10] Health Level Seven International (2023). FHIR Release 5: Overview. Fast Healthcare Interoperability Resources Specification.

[11] Parry M., Huang T., Clarke H., Bjørnnes A.K., Harvey P., Parente L., Norris C., Pilote L., Price J., Stinson J.N. (2025). Development and Systematic Evaluation of a Progressive Web Application for Women With Cardiac Pain: Usability Study. JMIR Cardio.

[12] Barão R.C., Hemelings R., Abegão Pinto L., Pazos M., Stalmans I. (2024). Artificial Intelligence for Glaucoma: State of the Art and Future Perspectives. Curr. Opin. Ophthalmol..

[13] Betzler B.K., Chen H., Cheng C.Y., Lee C.S., Ning G., Song S.J., Lee A.Y., Kawasaki R., van Wijngaarden P., Grzybowski A. (2023). Large Language Models and Their Impact in Ophthalmology. Lancet Digit. Health.

[14] Kleinig O., Gao C., Kovoor J.G., Gupta A.K., Bacchi S., Chan W.O. (2024). How to Use Large Language Models in Ophthalmology: From Prompt Engineering to Protecting Confidentiality. Eye.

[15] Agnihotri A.P., Nagel I.D., Artiaga J.C.M., Guevarra M.C.B., Sosuan G.M.N., Kalaw F.G.P. (2025). Large Language Models in Ophthalmology: A Review of Publications from Top Ophthalmology Journals. Ophthalmol. Sci..

[16] Tseng R.M.W.W., Gunasekeran D.V., Tan S.S.H., Rim T.H., Lum E., Tan G.S.W., Wong T.Y., Tham Y.C. (2021). Considerations for Artificial Intelligence Real-World Implementation in Ophthalmology: Providers’ and Patients’ Perspectives. Asia Pac. J. Ophthalmol..

[17] Yang W.-H., Shao Y., Xu Y.-W. (2023). Expert Workgroup of Guidelines on Clinical Research Evaluation of Artificial Intelligence in Ophthalmology (2023); Ophthalmic Imaging and Intelligent Medicine Branch of Chinese Medicine Education Association; Intelligent Medicine Committee of Chinese Medicine Education Association. Guidelines on Clinical Research Evaluation of Artificial Intelligence in Ophthalmology (2023). Int. J. Ophthalmol..

[18] World Health Organization (2021). Ethics and Governance of Artificial Intelligence for Health: WHO Guidance.

[19] Allen B., Seltzer S.E., Langlotz C.P., Dreyer K.P., Summers R.M., Petrick N., Marinac-Dabic D., Cruz M., Doraiswamy P.M., Jaffe T.A. (2019). A Road Map for Translational Research on Artificial Intelligence in Medical Imaging: From Laboratory to Clinical Practice. J. Am. Coll. Radiol..

[20] Brooke J., Jordan P.W., Thomas B., McClelland I.L., Weerdmeester B. (1996). SUS: A Quick and Dirty Usability Scale. Usability Evaluation in Industry.

[21] Bangor A., Kortum P., Miller J. (2009). Determining What Individual SUS Scores Mean: Adding an Adjective Rating Scale. J. Usability Stud..

[22] Lewis J.R., Sauro J. (2016). Quantifying the User Experience: Practical Statistics for User Research.

[23] Hyzy M., Bond R., Mulvenna M., Bai L., Dix A., Leigh S., Hunt S. (2022). System Usability Scale Benchmarking for Digital Health Apps: Meta-Analysis. JMIR mHealth uHealth.

[24] Gonçalves R.L., Pagano A.S., Reis Z.S.N., Brackstone K., Lopes T.C.P., Cordeiro S.A., Nunes J.M., Afagbedzi S.K., Head M., Meira W. (2023). Usability of Telehealth Systems for Noncommunicable Diseases in Primary Care from the COVID-19 Pandemic Onward: Systematic Review. J. Med. Internet Res..

[25] McDonald L., Glen F.C., Taylor D.J., Crabb D.P. (2017). Self-Monitoring Symptoms in Glaucoma: A Feasibility Study of a Web-Based Diary Tool. J. Ophthalmol..

[26] Wu P.C., Chiang W.Y., Lo J., Lee J.J., Chen Y.J., Kuo H.K., Chiau J.S., Hsu S.H., Chen Y.H. (2024). Using a Smartphone-Based Chatbot for Postoperative Care After Intravitreal Injection During the COVID-19 Pandemic: Retrospective Cohort Study. JMIR Form. Res..

[27] Radcliffe N.M., Shah M., Samuelson T.W. (2023). Challenging the “Topical Medications-First” Approach to Glaucoma: A Treatment Paradigm in Evolution. Ophthalmol. Ther..

[28] Sproul A., Stevens J., Richard J. (2023). Older Adults’ Use of and Interest in Technology and Applications for Health Management: A Survey Study. Can. J. Hosp. Pharm..

[29] Félix J., Moreira J., Santos R., Kontio E., Pinheiro A.R., Sousa A.S.P. (2023). Health-Related Telemonitoring Parameters/Signals of Older Adults: An Umbrella Review. Sensors.

[30] Siette J., Dodds L., Sharifi F., Nguyen A., Baysari M., Seaman K., Raban M., Wabe N., Westbrook J. (2023). Usability and Acceptability of Clinical Dashboards in Aged Care: Systematic Review. JMIR Aging.

[31] Zou C., Harvard A., Qian J., Fox B.I. (2023). A Systematic Review of Digital Health Technologies for the Care of Older Adults During the COVID-19 Pandemic. Digit. Health.

[32] Kruse C., Fohn J., Wilson N., Nunez Patlan E., Zipp S., Mileski M. (2020). Utilization Barriers and Medical Outcomes Commensurate With the Use of Telehealth Among Older Adults: Systematic Review. JMIR Med. Inform..

